# Task Shifting Routine Inpatient Pediatric HIV Testing Improves Program Outcomes in Urban Malawi: A Retrospective Observational Study

**DOI:** 10.1371/journal.pone.0009626

**Published:** 2010-03-10

**Authors:** Eric D. McCollum, Geoffrey A. Preidis, Mark M. Kabue, Emmanuel B. M. Singogo, Charles Mwansambo, Peter N. Kazembe, Mark W. Kline

**Affiliations:** 1 Baylor International Pediatric AIDS Initiative, Baylor College of Medicine, Lilongwe, Malawi; 2 Interdepartmental Program in Translational Biology and Molecular Medicine, Department of Pediatrics, Baylor College of Medicine, Houston, Texas, United States of America; 3 Baylor Children's Foundation-Malawi, Baylor College of Medicine, Lilongwe, Malawi; 4 Department of Pediatrics, Kamuzu Central Hospital, Lilongwe, Malawi; 5 Baylor International Pediatric AIDS Initiative, Baylor College of Medicine, Houston, Texas, United States of America; University of Cape Town, South Africa

## Abstract

**Background:**

This study evaluated two models of routine HIV testing of hospitalized children in a high HIV-prevalence resource-constrained African setting. Both models incorporated “task shifting,” or the allocation of tasks to the least-costly, capable health worker.

**Methods and Findings:**

Two models were piloted for three months each within the pediatric department of a referral hospital in Lilongwe, Malawi between January 1 and June 30, 2008. Model 1 utilized lay counselors for HIV testing instead of nurses and clinicians. Model 2 further shifted program flow and advocacy responsibilities from counselors to volunteer parents of HIV-infected children, called “patient escorts.” A retrospective review of data from 6318 hospitalized children offered HIV testing between January-December 2008 was conducted. The pilot quarters of Model 1 and Model 2 were compared, with Model 2 selected to continue after the pilot period. There was a 2-fold increase in patients offered HIV testing with Model 2 compared with Model 1 (43.1% vs 19.9%, p<0.001). Furthermore, patients in Model 2 were younger (17.3 vs 26.7 months, p<0.001) and tested sooner after admission (1.77 vs 2.44 days, p<0.001). There were no differences in test acceptance or enrollment rates into HIV care, and the program trends continued 6 months after the pilot period. Overall, 10244 HIV antibody tests (4779 maternal; 5465 child) and 453 DNA-PCR tests were completed, with 97.8% accepting testing. 19.6% of all mothers (n = 1112) and 8.5% of all children (n = 525) were HIV-infected. Furthermore, 6.5% of children were HIV-exposed (n = 405). Cumulatively, 72.9% (n = 678) of eligible children were evaluated in the hospital by a HIV-trained clinician, and 68.3% (n = 387) successfully enrolled into outpatient HIV care.

**Conclusions/Significance:**

The strategy presented here, task shifting from lay counselors alone to lay counselors and patient escorts, greatly improved program outcomes while only marginally increasing operational costs. The wider implementation of this strategy could accelerate pediatric HIV care access in high-prevalence settings.

## Introduction

Globally, an estimated 2.2 million children younger than 15 years of age were HIV-infected (HIV+) in 2007, with 88% living in sub-Saharan Africa [1]. Malawi, a sub-Saharan African country of 13.2 million people, reports an adult HIV prevalence of 11.9% [1]. While 91,000 HIV+ children were estimated to be living in Malawi in 2007 [2], less than 8% of all Malawian children have ever been HIV tested [3]. Additionally, HIV+ children comprised just 8% of all national antiretroviral therapy (ART) recipients, below the 2014 goal of 15% [3]. To address this gap in HIV care, Malawi approved the routine offering of HIV testing, defined here as provider initiated HIV testing and counseling (PITC), in line with global recommendations in 2007 [4]. A high HIV prevalence in hospitalized Malawian children has been reported [5]–[8]; however, current guidelines do not offer a strategy for pediatric hospital-based PITC in resource-constrained settings [4].

In 2005, the Baylor College of Medicine-Abbott Fund Children's Clinical Centre of Excellence (COE) established a pediatric HIV program in Malawi that included patient care, national capacity building, and technical assistance [9], [10]. The COE's outpatient clinic is located adjacent to the Kamuzu Central Hospital (KCH) pediatric inpatient department and has enrolled over 4200 patients to date [Baylor International Pediatric AIDS Initiative, unpublished data]. KCH is a 215 pediatric bed referral hospital located in the capital of Lilongwe with more than 13000 pediatric admissions annually. Severe nurse and clinician shortages strain hospital systems and contribute to pediatric mortality rates that exceed 8% [KCH, unpublished data].

In January 2008, the COE and KCH implemented a PITC program in the pediatric department. In a 24 month period preceding the PITC program, 1613 total children accessed non-routine HIV counseling and testing services offered by lay counselors in the pediatric ward, representing less than 6% of all pediatric inpatients [11]. The PITC program's objectives were the routine offering of confidential and timely HIV testing, enrolling HIV+ and HIV-exposed (HIV-E) hospitalized children into HIV care, and establishing a scalable model.

“Task shifting,” or the allocation of tasks to the least costly, capable health worker, is an accepted approach to addressing human resource constraints [12]-[17]. Our program sequentially piloted two PITC models that utilized different task shifting strategies. Model 1 continued to use lay counselors for HIV testing rather than nurses and clinicians. Model 2 shifted program flow and advocacy tasks from counselors to volunteer parents of HIV+ children, called “patient escorts,” while continuing to utilize counselors for testing. One COE clinician provided daily HIV care for all hospitalized HIV+ and HIV-E children. Inpatient care included the prescription of co-trimoxazole preventative therapy, treatment of opportunistic infections, supportive care, and outpatient referral. Outpatient care at the COE consisted of free, comprehensive pediatric HIV services, including ART and co-trimoxazole provision by COE clinicians.

The feasibility, acceptability, and referral of PITC-identified outpatients to HIV care in resource-limited settings have been demonstrated [18]–[28]. While a referral hospital in Zambia recently described a routine inpatient pediatric HIV testing program using lay counselors [19], no studies to date have investigated whether alternate strategies using volunteers rather than additional professional staff can achieve similar programmatic outcomes while only marginally increasing operational expenses. We hypothesized that a model incorporating lay counselors and additional volunteers would more efficiently achieve this program's objectives in an under-resourced, overburdened hospital.

## Methods

### Program Implementation and Description

In 2007 a multidisciplinary task force at KCH created a PITC program strategy, program procedural manual [Text S1], and confidential program register [Text S2]. The task force also revised the pediatric department admissions form to include a standing written order for HIV testing as well as a section for documentation of test results. Based on low nurse and clinician staffing levels, high rates of evening admissions when staffing was even lower, and undeveloped triage systems, the task force concluded that HIV testing at admission by nurses and clinicians could not achieve the program objectives and could delay emergent care. Furthermore, the task force decided that a bedside testing model was potentially coercive and inadequate for confidentiality. Instead, the group concluded that a model built around private testing by counselors during inpatient ward rounds, with or without patient escorts, could be immediately piloted.

A program algorithm divided PITC into eight steps [Text S3], and the confidential patient register facilitated referrals and monitoring [Text S2]. All PITC staff worked from 7:30am to 4:00pm Monday through Friday. HIV testing was not available on weekends. Government approval was obtained, and funding was acquired from the Baylor International Pediatric AIDS Initiative. Baylor International Pediatric AIDS Initiative receives primary funding from Bristol Myers Squibb, the Abbott Fund, Texas Children's Hospital, The United Nations Children's Fund, and the Malawi Ministry of Health.

Two rooms within the pediatric department were equipped with partitions and materials to create four private testing spaces. Four nationally certified HIV counselors employed by the COE and Lighthouse Trust were assigned to the program, with one counselor per room. Prior to implementing each model, two half-day departmental orientations took place each preceding month [Text S4, S5, S6]. As a part of the orientation, clinicians and nurses were trained to order HIV testing on all patients during wards rounds, irrespective of clinical symptoms, physical examination, admission diagnosis, or ward location. Counselors also received guidance regarding how to conduct pretest group counseling and opt-out testing.

In both models, counselors followed a PITC opt-out protocol and the Malawi National HIV Counseling and Testing guidelines. National guidelines recommended investigating maternal HIV status before testing the child. Eligible children were tested with a HIV antibody test and/or DNA-PCR test depending upon their age [29]. In some select cases, however, the child was tested first, and the mother was either tested after the child or not at all. If the caregiver produced valid documentation, such as the national health passport, indicating that the child's HIV status was known, then testing information was documented on the patient's admission form and the PITC register was updated. Patients that disclosed their HIV status during ward rounds would not go to the counseling room for testing. Two COE clinicians evaluated all hospitalized HIV+ and HIV-E children using the confidential program register to locate patients, and provided program supervision. Supervision consisted of a weekly review of program registers and monthly PITC meetings within the pediatric department. The meetings provided monitoring feedback to staff and addressed programmatic or staff issues.

The Model 1 pilot period began January 1, 2008 and continued until March 31, 2008. In addition to opt-out HIV testing, Model 1 expected counselors to perform group counseling, team up with providers during ward rounds, interact with patients hospitalized on the ward, and accompany caregivers to the testing rooms.

Four patient escorts were identified for Model 2 and introduced at the Model 2 orientation. The escorts were recruited from a group of pro-active parents of HIV+ children that were attending the COE clinic. Each escort was openly HIV+, compliant with ART, demonstrated proficient oral and written English and Chichewa language skills, and produced documentation confirming completion of primary schooling. All escorts received a half-day orientation [Text S4, S5, S6], as well as pediatric HIV diagnosis [Text S7, S8, S9, S10, S11, S12] and job-related training [Text S13, Text S14], followed by one week of on-the-job supervision by a COE pediatrician. Each volunteer patient escort earned a stipend for work-related transportation and food ($2/day).

The Model 2 pilot period began April 1, 2008 and continued until June 30, 2008. Each escort was assigned to a counselor and KCH clinician for ward rounds, and accompanied patients to the testing room after a provider advised the caregiver about the routine HIV test. If the providers were unavailable or did not advise the caregiver appropriately, escorts would still mentor parents regarding the benefits of pediatric HIV testing and chaperone willing caregivers to the counseling room. Counselors continued to perform group counseling and opt-out HIV testing.

The PITC task force evaluated the pilot models, and selected Model 2 for the programmatic period that started July 1, 2008.

### Study Design

The study was approved by the Malawi National Health Sciences Research Committee and Baylor College of Medicine institutional review boards, respectively. Verbal consent for HIV testing was obtained by nationally certified HIV counselors and documented in patient files in accordance with Malawi National HIV Counseling and Testing guidelines [29]. Written consent was not required by study participants since data was collected as a part of routine program monitoring and evaluation. A retrospective review of data from 6318 hospitalized children offered HIV testing from January-December 2008 was conducted. The review was separated into two periods, pilot and programmatic. Disaggregated pre-PITC pediatric ward HIV testing data was unavailable for comparison. A data officer collected maternal and child study data from the PITC register, ward admissions book, and COE electronic medical record. Outcomes data included the proportion of admissions offered HIV testing, the proportion accepting testing, age in months of PITC recipients, days elapsed from admission to testing, maternal HIV antibody test results, child HIV antibody and DNA-PCR test results, mother and child HIV status, and successful enrollment into inpatient and outpatient pediatric HIV care.

### Statistical Analysis

Continuous variables were evaluated with the Mann-Whitney U test. Age and time from hospital admission to HIV test were expressed as median months and mean days, respectively, both with interquartile range. For categorical parameters, data were reported as raw value and percentage of the respective group. Pearson's chi-square test was used to determine global significance between Model 1 and Model 2 pilot period data, and post-hoc analyses were performed using pair-wise chi-square tests or Fisher exact tests, with the Bonferroni correction applied to adjust α for multiple variable levels. Only p values smaller than the corrected α were considered statistically significant. All statistical analyses were performed using SPSS software (version 17.0; SPSS Inc., Chicago, IL).

## Results

During the one-year study period, 13981 children were admitted to KCH. Overall, 45.2% of children were offered HIV testing, with a 2-fold increase in proportion observed during the Model 2 pilot period (43.1%), compared to Model 1 (19.9%). The HIV testing rate further increased to 64.5% during the programmatic period. “Test offering” was defined as patients offered HIV testing by a clinician or nurse, lay counselor or patient escort within the ward, or by a lay counselor within the testing room. Test acceptance exceeded 96% throughout the pilot and programmatic periods, with no differences observed between the models. Characteristics of children offered testing are described in Table 1. Compared to the Model 1 pilot quarter, children tested during the Model 2 pilot period were 9.4 months younger, with a more than one-half day reduction in the time between hospital admission and an HIV test. These trends continued through the programmatic period. While there was a decrease in the proportion of children previously HIV tested between Model 1 (4.4%) and the pilot quarter of Model 2 (2.2%), a 7-fold increase to 15.5% of children previously HIV tested was observed between the Model 2 programmatic and pilot period. No gender differences were observed between the two models.

**Table 1:** PITC Pediatric Inpatient Characteristics.

Characteristic	Pilot Period Model 1 Jan-Mar 2008	Pilot Period Model 2 Apr-Jun 2008	Programmatic Period Model 2 Jul-Dec 2008	Total Jan-Dec 2008	*p*-Value^1^
Pediatric admissions offered HIV test, *n/N* (%)	830/4172 (19.9)	1689/3919 (43.1)	3799/5890 (64.5)	6318/13981 (45.2)	<0.001
Children offered HIV test with unknown HIV status,^2^ *n/N* (%)	793/830 (95.5)	1653/1689 (97.9)	3211/3799 (84.5)	5657/6318 (89.5)	0.001
HIV test acceptance, *n/N* (%)	787/793 (99.2)	1639/1653 (99.2)	3105/3211 (96.7)	5531/5657 (97.8)	0.816
Time from child's admission to HIV test, d, mean (IQR)	2.44 (1–3)	1.77 (1–2)	1.61 (1–2)	1.76 (1–2)	<0.001
Child's age, mos, median (IQR)	26.7 (12.2–50.0)	17.3 (8.7–35.3)	15.8 (7.2–36.0)	17.4 (8.1–37.7)	<0.001
Females, *n/N* (%)	412/830 (49.6)	825/1689 (48.8)	1865/3799 (49.1)	3102/6318 (49.1)	0.708

1 Pilot Period Model 1 × Pilot Period Model 2; Pearson chi-square test for categorical variables; Mann-Whitney U test for continuous variables.

2 The patient was not re-tested if they disclosed a documented and valid previous HIV test result. During Pilot Period Model 1, no children offered HIV testing were readmissions with a known HIV status. During Pilot Period Model 2, 5 (0.3%) children offered HIV testing were readmissions with a known HIV status. During Programmatic Period Model 2, 102 (2.7%) children offered HIV testing were readmissions with a known HIV status.

**Figure 1:**
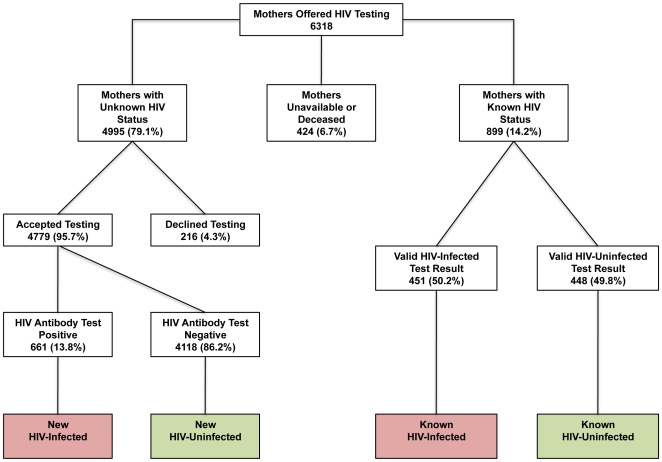
Maternal PITC Overview at Kamuzu Central Hospital; January 1-December 31, 2008. Total HIV-infected mothers (red boxes): 1112/5678 (19.6%); total HIV-uninfected mothers (green boxes): 4566/5678 (80.4%).

HIV antibody and DNA-PCR testing, if applicable, was completed on hospitalized children, and on consenting mothers in accordance with national HIV testing guidelines [29]. Overall, 10244 HIV antibody tests (4779 maternal; 5465 child) and 453 HIV DNA-PCR tests were completed. No test stock-outs were experienced. Maternal test results and HIV status are shown in Table 2 with an overview provided in Figure 1. Cumulatively, 19.6% of mothers were HIV+, with more mothers testing HIV-uninfected during the Model 2 pilot period. Importantly, Model 2 significantly increased the proportion of mothers with an unknown HIV status offered HIV testing. 7.2% of mothers disclosed a valid previous test result during both periods.

**Table 2:** Maternal HIV Antibody Test Results and HIV Status.

Characteristic	Category	Pilot Period Model 1 Jan-Mar 2008^2^	Pilot Period Model 2 Apr-Jun 2008^3^	Programmatic Period Model 2 Jul-Dec 2008^4^	Total Jan-Dec 2008	*p*-Value^1^
Mothers offered HIV test with unknown HIV status, *n/N* (%)		625/830 (75.3)	1442/1689 (85.4)	2712/3799 (71.4)	4995/6318 (79.1)	<0.001
HIV test acceptance, *n/N* (%)		610/625 (97.6)	1412/1442 (97.9)	2541/2712 (93.7)	4779/4995 (95.7)	0.647
Maternal HIV antibody test, *n/N* (%)						0.001
	Positive	120/625 (19.2)	193/1442 (13.4)	348/2712 (12.8)	661/4779 (13.8)	
	Negative	505/625 (80.8)	1249/1442 (86.6)	2364/2712 (87.2)	4118/4779 (86.2)	
Maternal HIV status^5^, *n/N* (%)						0.024
	HIV-infected	141/685 (20.6)	260/1563 (16.6)	711/3430 (20.7)	1112/5678 (19.6)	
	HIV-uninfected	544/685 (79.4)	1303/1563 (83.4)	2719/3430 (79.3)	4566/5678 (80.4)	

1 Pilot Period Model 1 × Pilot Period Model 2; Pearson chi-square test.

2 During Pilot Period Model 1, 130 (15.7%) mothers were either unavailable for HIV antibody testing or deceased, 15 (1.8%) declined HIV testing, and 21 (2.5%) and 39 (4.7%) mothers were not retested as they were known HIV-infected and HIV-uninfected, respectively, after disclosing a documented and valid previous HIV test result.

3 During Pilot Period Model 2, 96 (5.7%) mothers were either unavailable for HIV antibody testing or deceased, 30 (1.8%) declined HIV testing, and 67 (4.0%) and 54 (3.2%) mothers were not retested as they were known HIV-infected and HIV-uninfected, respectively.

4 During Programmatic Period Model 2, 198 (5.2%) mothers were either unavailable for HIV antibody testing or deceased, 171 (4.5%) declined HIV testing, and 363 (9.6%) and 355 (9.3%) mothers were not retested as they were known HIV-infected and HIV-uninfected, respectively.

5 Includes newly identified and previously known HIV-infected and HIV-uninfected mothers.

**Figure 2:**
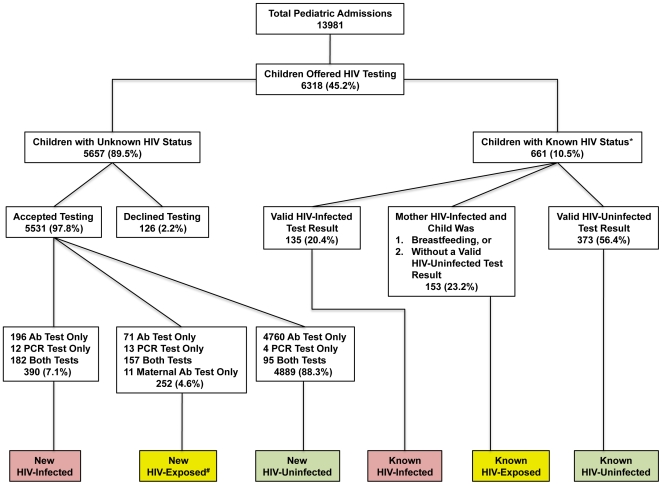
Child PITC Overview at Kamuzu Central Hospital; January 1 - December 31, 2008. Total HIV-infected children (red boxes): 525/6192 (8.5%); total HIV-exposed children (yellow boxes): 405/6192 (6.5%); total HIV-uninfected children (green boxes): 5262/6192 (85.0%). *6 children with known HIV status were incorrectly retested. ^#^158 newly recognized HIV-exposed children had a negative DNA-PCR while breastfeeding, while 12 DNA-PCR results for newly recognized HIV-exposed children were lost. Ab: HIV antibody; PCR: HIV DNA-PCR.

Child HIV antibody and DNA-PCR test results are reported in Table 3, and child HIV status is detailed in Table 4. Figure 2 provides an overview of child HIV testing. Overall, 8.5% of children were HIV+ and 6.5% were HIV-E, defined as a child born to an HIV+ mother who was either without a definitive HIV test result or who was DNA-PCR negative but still breastfeeding. While there were no significant differences in HIV-E status between the models, a higher percentage of HIV-E patients were observed during the programmatic period.

**Table 3:** Child HIV Test Results.

Characteristic	Category	Pilot Period Model 1 Jan-Mar 2008	Pilot Period Model 2 Apr-Jun 2008	Programmatic Period Model 2 Jul-Dec 2008	Total Jan-Dec 2008	*p*-Value^1^
Child HIV antibody test, *n/N* (%)						0.001
	Positive	91/776 (11.7)	126/1633 (7.7)	210/3056 (6.9)	427/5465 (7.8)	
	Negative	685/776 (88.3)	1507/1633 (92.3)	2847/3056 (93.1)	5039/5465 (92.2)	
Child HIV DNA PCR test, *n/N* (%)						0.082
	Positive	42/90 (46.7)	47/134 (35.1)	105/229 (45.9)	194/453 (42.8)	
	Negative	48/90 (53.3)	87/134 (64.9)	124/229 (54.1)	259/453 (57.2)	

1 Pilot Period Model 1 × Pilot Period Model 2; Pearson chi-square test.

**Table 4:** Child HIV Status.

Category	Sub-Category	Pilot Period Model 1 Jan-Mar 2008	Pilot Period Model 2 Apr-Jun 2008	Programmatic Period Model 2 Jul-Dec 2008	Total Jan-Dec 2008	*p*-Value^1^
HIV-infected, *n/N* (%)		97/824 (11.8)	108/1675 (6.4)	320/3693 (8.7)	525/6192 (8.5)	0.203
	New	92/97 (94.8)	97/108 (89.8)	201/320 (62.8)	390/525 (74.3)	
	Known^2^	5/97 (5.2)	11/108 (10.2)	119/320 (37.2)	135/525 (25.7)	
HIV-exposed, *n/N* (%)		32/824 (3.9)	98/1675 (5.9)	275/3693 (7.4)	405/6192 (6.5)	1.000
	New	30/32 (93.8)	93/98 (94.9)	129/275(46.9)	252/405 (62.2)	
	Known^2^	2/32(6.3)	5/98 (5.1)	146/275 (53.1)	153/405 (37.8)	
HIV-uninfected, *n/N* (%)		695/824 (84.3)	1469/1675 (87.7)	3098/3693 (83.9)	5262/6192 (85.0)	<0.001
	New	665/695 (95.7)	1449/1469 (98.6)	2775/3098 (89.6)	4889/5262 (92.9)	
	Known^2^	30/695 (4.3)	20/1469 (1.4)	323/3098 (10.4)	373/5262 (7.1)	

1 Pilot Period Model 1 × Pilot Period Model 2; Pearson chi-square test or Fisher's exact test.

2 The patient was not re-tested, as they disclosed a documented and valid previous HIV test result.

Table 5 shows the proportion of children that successfully enrolled into inpatient and outpatient pediatric HIV care. All successfully referred inpatient HIV+ and HIV-E hospitalized children were evaluated by a COE clinician, and were referred to an outpatient HIV clinic at discharge. All patients that received a DNA-PCR, however, were expected to return to the COE for their results whether or not a COE clinician reviewed them during their hospitalization. Overall, inpatient HIV clinicians reviewed 72.9% of HIV+ and HIV-E children during their hospital admission. Of those not previously enrolled into HIV care, 567 accepted outpatient referral to the COE with 68.3% successfully returning and enrolling into care at the clinic. There were no differences in successful inpatient and outpatient pediatric HIV care enrollment rates between the two models.

**Table 5:** Inpatient and Outpatient Pediatric HIV Care Enrollment.

Characteristic	Pilot Period Model 1 Jan-Mar 2008	Pilot Period Model 2 Apr-Jun 2008	Programmatic Period Model 2 Jul-Dec 2008	Total Jan-Dec 2008	*p*-Value^1^
Successful inpatient enrollment, *n/N* (%)	85/128 (66.4)	155/214 (72.4)	438/588 (74.5)	678/930 (72.9)	0.239
Successful outpatient enrollment, *n/N* (%)	75/101 (74.3)	100/147 (68.0)	212/319 (66.5)	387/567 (68.3)	0.290

1 Pilot Period Model 1 × Pilot Period Model 2; Pearson chi-square test.

## Discussion

This report reveals that a routine inpatient pediatric HIV testing model incorporating lay counselors and volunteer patient escorts significantly increased the number of children offered testing, maintained high acceptance rates, and shortened the time between hospital admission and HIV testing. These findings suggest that at this crowded, resource-constrained sub-Saharan African hospital, four counselors alone were insufficient to achieve the PITC program objectives. Rather than hire additional counselors and significantly increase operational costs, we improved performance with only a marginal increase in expenses by task-shifting program advocacy and patient flow responsibilities to four capable volunteer patient escorts.

Before the implementation of our model of PITC, clinicians and nurses non-routinely offered HIV testing within the pediatric inpatient wards based upon a patient's clinical findings, otherwise patients independently accessed the service themselves. Less than 6% of pediatric inpatients received HIV tests utilizing this strategy [11].

There are multiple reasons why few inpatients accessed HIV testing before PITC, and also during the Model 1 Pilot Period. The high ratio of patients to providers in the pediatric ward (>25 patients to 1 provider) made it difficult for any clinician or nurse to dedicate the time needed to order HIV testing and continue their other duties. Providers also expressed hesitancy to order testing because they perceived the process as requiring an extensive, specialized counseling session, which they might have felt inadequately trained to convey. Few providers felt comfortable with pediatric HIV care in general, viewing it as a condition for specialists only, and few appreciated the value of knowing a patient's HIV status during the hospitalization. Furthermore, severe human resource constraints and poor retention of pediatric ward staff forced administrators to rely upon temporarily employed clinicians and nurses to provide a majority of patient care. This lack of provider continuity limited the effectiveness of a system that heavily relied upon this cadre of healthcare workers.

While most caregivers readily accepted HIV testing, a minority of parents would require more intensive counseling. These parents expressed a reluctance to go to the testing room for a variety of reasons, including HIV-related stigma, the absence of their spouse's approval, a fear of missing nursing medication rounds, a concern that their child was “too sick” to be tested, a lack of awareness that children could benefit from ART, or a feeling of intimidation by the chaotic, overwhelming hospital environment itself. Thus, for a subset of patients the provider did need to dedicate more time to impart bedside counseling and accompany the patient to the counseling room for testing. Unfortunately, providers rarely made this effort. Furthermore, HIV counselors did little to support providers as they remained inside the HIV testing room and did not actively engage patient caregivers, nurses, or clinicians working on the ward.

While these barriers continued to persist during our intervention, they provide critical insight to explain why patient escorts dramatically improved our program's outcomes, and why Model 1 was only marginally successful. Model 1 expected counselors to proactively collaborate with providers, routinely engage patients at the bedside, and chaperone caregivers to the testing rooms. The contributions of the counselors were inconsistent at best. While the counselors that were willing and motivated routinely succeeded, the patient volume and demand for testing overwhelmed the system.

Patient escorts effectively bridged the gaps that providers and counselors were unable or unwilling to fill. Compared to providers, the volunteers could spend more time talking with caregivers about the benefits of testing, often motivating them with personal stories. When providers were either unavailable or did not discuss HIV testing with the caregiver, the patient escorts successfully advocated testing to almost all patients, and would subsequently accompany them to the testing room. The escorts also increased the efficiency of the counselors, allowing them to focus upon their more familiar tasks of testing and counseling in their private room rather than managing patient flow on the ward.

Over time, the patient escorts' skills improved and the providers and counselors accepted their contributions warmly. This observation is supported by the continued progress demonstrated during the programmatic period when 64.5% of pediatric admissions were offered HIV testing and the number of patients disclosing a valid, previously-known HIV status greatly increased. The latter observation, including a 7-fold increase in those known to be HIV+, an 8-fold increase in children known to be HIV-E, and a more than 2-fold increase in those known to be HIV-uninfected, is a notable achievement. Model 2 thus fulfills a vital program objective, as it improves efficiency by reducing unnecessary retesting and ensures that all patients have the opportunity to be reviewed by clinicians after consideration of their HIV status.

There are several limitations to the model presented here. This program relied upon private testing space within the inpatient ward. While this approach maximized discretion and confidentiality, other hospitals may not have space available and would need to create space or modify this strategy. Furthermore, counselors did not staff the department during the weekend, which reduced testing availability. These limitations might be costly to address in many resource-constrained hospitals. While the lack of weekend HIV testing was an identifiable program drawback, these data collection tools were not designed to gather patient-level information about children not offered HIV testing, thus precluding analysis of whether any groups were routinely omitted. This drawback limits the usefulness of our data for detecting program weaknesses. However, when weekend admissions during the programmatic period were excluded, the proportion of pediatric admissions offered testing reached 89.6%.

In addition, the sequential implementation of the models could have biased outcomes in favor of Model 2. However, the immediate two-fold increase and sustained HIV testing levels during the programmatic period more likely reflects the impact of the patient escorts rather than the three additional months of PITC implementation. While the possibility exists that under-reporting of declined HIV testing could have contributed to the high test acceptance rates, it is unlikely that the rates reflect coercion, as this was monitored closely and not detected during unannounced observation visits by a COE pediatrician. Furthermore, a selection bias regarding outpatient care referral is possible, as we were unable to track the proportion of children that enrolled into outpatient HIV care at sites other than the COE. However, all patients, regardless of their follow-up preferences, were provided the same standard of inpatient HIV care and were counseled about outpatient follow-up similarly. Lastly, because no reliable pre-intervention data was available, we were unable to statistically compare either model with the previous HIV testing and counseling approach.

The median length of hospitalization in the KCH general pediatric ward was 3.0 days during both pilot quarters (data not shown), which implies that seasonal changes in the duration of hospitalization did not preferentially bias the increased HIV testing proportion observed in Model 2. Importantly, this program's test acceptance rate compares favorably to other reports [20]-[25], suggesting that if a similar model were brought to scale in Malawi, the proportion of children ever HIV tested would far surpass current estimates [3]. Importantly, as a substantial proportion of Malawian children die within 2 days of hospital admission, the reduction in time elapsed between hospital admission and HIV testing observed during Model 2 could also allow clinicians to consider a patient's HIV status earlier, provide more directed care for opportunistic infections and other complicating conditions sooner, and improve hospital survival [30].

While the overall proportions of patients enrolled into HIV care were comparable to other published reports [21]–[23], [25], [26], this program's 72.9% inpatient and 68.3% outpatient care enrollment rates could be further improved. Of note, these proportions represent actual HIV care received, not simply referral statistics, which are more commonly reported. Programmatic weaknesses that may have reduced these rates include premature hospital discharge by KCH clinicians, inadequate patient defaulter tracking, high transportation costs, weak relationships with community health centers, poor parental understanding of the needs of chronically ill children, and continued HIV-related stigma. Despite the barriers challenging enrollment of any HIV+ child into care, the rates of care demonstrated in this study suggest that pediatric ART recipients, as a percentage of total ART recipients, could more closely approach Malawi's 2014 goal of 15% with broader implementation of this strategy.

Substantial percentages of HIV+ infants suffer from high mortality without early infant diagnosis (EID) and ART initiation irrespective of CD4% [31]–[36]. Malawi implemented universal ART for all HIV+ children younger than 1 year of age in 2008, highlighting the need to further strengthen EID services with programs that test younger children for HIV. Importantly, Model 2, which incorporated patient escorts, significantly reduced the age of children HIV tested. Cumulatively, hospitalized children at KCH were 2.9 months younger during the Model 2 pilot period compared to Model 1, likely due to documented seasonal increases in lower respiratory tract infection, malaria, and diarrhea at KCH (data not shown). However, this difference does not fully account for the observed 9.4 month median age reduction in children offered HIV testing from Model 1 to Model 2, a reduction that continued during the programmatic period. With wider implementation, the strategy outlined in this report could strengthen EID through the identification and enrollment into care of significantly more HIV+ children younger than 1 year of age, and of more children earlier in their clinical course of disease.

Kankasa et al recently demonstrated that routine HIV testing of children admitted to a referral hospital in Lusaka, Zambia was feasible and acceptable, using a bedside HIV antibody testing strategy that utilized higher numbers of counselors [19]. Our results confirm the acceptability of routine inpatient pediatric HIV testing in a similar setting in Malawi. However, hiring additional nationally certified HIV counselors would have prohibitively increased operational costs for our program, and was determined by the PITC task force to be infeasible at KCH. The task force opted instead to limit the number of paid counselors to four, and to determine whether volunteer patient escorts could improve PITC outcomes compared to lay counselors alone. Furthermore, our overall test acceptance rate of 97.8%, compared to the 87.4% reported by Kankasa et al. [19], suggests that testing performed in a private room could be less stigmatizing than bedside testing in this setting.

This study's overall 8.5% pediatric inpatient HIV prevalence is nearly double the estimated national 4.8% pediatric prevalence reported in 2008 [3], but is less than the 18.9% prevalence reported by Rogerson et al from Blantyre, Malawi in 2000 [5]. The higher prevalence observed by Rogerson et al likely reflects the absence of free public sector ART services at the time of their study as well as differences in HIV prevalence between the southern (i.e. Blantyre) and central regions (i.e. Lilongwe) [37]. Few reports cite the percentage of hospitalized HIV-E children in sub-Saharan Africa. This study's observed 6.5% HIV-E status suggests that many HIV-E children were accessing hospital care without inpatient identification and referral. The hospitalization of this cohort represents a critical opportunity for further prevention of mother-to-child transmission interventions including ongoing breastfeeding counseling [38]–[43] and initiation of extended maternal and infant post-natal antiretroviral prophylaxis regimens [43]–[46].

At the time of this study, routine HIV care services in Malawi were primarily restricted to children receiving ART rather than ART-ineligible or HIV-E children. Overall, this study identified a high proportion of hospitalized HIV+ and HIV-E children that were previously identified, suggesting that these children might not be accessing HIV care services after testing or that their quality of outpatient care may be less than adequate. Both scenarios could result in HIV progression requiring hospitalization. Importantly, only 27 HIV+ and 19 HIV-E patients were identified as readmissions during the 12 month study period (data not shown), confirming that most hospital admissions of HIV+ and HIV-E patients were children new to KCH. These findings imply that pediatric HIV and prevention of mother-to-child transmission services must strengthen the linkage between testing and care, and that the hospital is an important care entry point for these patients.

Studies from Uganda and Zambia concluded that inpatient PITC programs provide an ongoing opportunity for HIV testing and care for entire families [19], [20]. Our study further supports these conclusions, as 4779 mothers received HIV testing, 13.8% of whom tested positive. While this program did not track outpatient referrals or HIV care enrollment of newly identified HIV+ mothers, we recommended that mothers be evaluated for ART eligibility at their child's outpatient appointment. We also recommended that other undiagnosed siblings attend the outpatient appointment for HIV testing.

In conclusion, the routine inpatient pediatric HIV testing strategy presented here, which incorporates task shifting from lay counselors alone to lay counselors and locally available patient escorts, greatly improved program outcomes while only marginally increasing operational expenses. Based on these results, we recommend that high HIV-prevalence countries suffering from severe human resource limitations implement similar programs as a core component of their national pediatric HIV care strategy. Further studies investigating the characteristics and outcomes of children identified via routine hospital-based HIV testing, counseling quality assessments, patient satisfaction surveys, cost modeling comparisons of alternate routine HIV testing strategies, and further head-to-head studies investigating different inpatient HIV testing approaches in urban and rural hospitals would add additional key information to this critically important area of pediatric HIV care.

## Supporting Information

Text S1 Inpatient Pediatric PITC Program - Manual of Procedures. A detailed description of inpatient pediatric PITC program procedures, recommended staffing levels, suggested budget, and program materials.
(8.80 MB TIF)

Text S2 Inpatient Pediatric PITC Program - Register. A confidential inpatient pediatric PITC program register for routine program monitoring and linkage of HIV-infected and HIV-exposed patients to inpatient clinical care.
(1.66 MB TIF)

Text S3 Inpatient Pediatric PITC Program - Overview. A one page overview outlining the eight steps that comprise the Baylor International Pediatric AIDS Initiative inpatient pediatric PITC system.
(0.26 MB TIF)

Text S4 Inpatient Pediatric PITC Program - Orientation. Pediatric department and inpatient pediatric PITC staff orientation presentation. Baylor International Pediatric AIDS Initiative PowerPoint slides 1–7.
(9.77 MB TIF)

Text S5 Inpatient Pediatric PITC Program - Orientation. Pediatric department and inpatient pediatric PITC staff orientation presentation. Baylor International Pediatric AIDS Initiative PowerPoint slides 8–13.
(9.57 MB TIF)

Text S6 Inpatient Pediatric PITC Program - Orientation. Pediatric department and inpatient pediatric PITC staff orientation presentation. Baylor International Pediatric AIDS Initiative PowerPoint slides 14–20.
(8.73 MB TIF)

Text S7 Diagnosis of Pediatric HIV Infection Training. Presentation to train the inpatient pediatric PITC program staff in pediatric HIV diagnosis. Baylor International Pediatric AIDS Initiative PowerPoint slides 1–6.
(7.67 MB TIF)

Text S8 Diagnosis of Pediatric HIV Infection Training. Presentation to train the inpatient pediatric PITC program staff in pediatric HIV diagnosis. Baylor International Pediatric AIDS Initiative PowerPoint slides 7–12.
(6.95 MB TIF)

Text S9 Diagnosis of Pediatric HIV Infection Training. Presentation to train the inpatient pediatric PITC program staff in pediatric HIV diagnosis. Baylor International Pediatric AIDS Initiative PowerPoint slides 13–18.
(7.75 MB TIF)

Text S10 Diagnosis of Pediatric HIV Infection Training. Presentation to train the inpatient pediatric PITC program staff in pediatric HIV diagnosis. Baylor International Pediatric AIDS Initiative PowerPoint slides 19–24.
(7.81 MB TIF)

Text S11 Diagnosis of Pediatric HIV Infection Training. Presentation to train the inpatient pediatric PITC program staff in pediatric HIV diagnosis. Baylor International Pediatric AIDS Initiative PowerPoint slides 25–29.
(6.51 MB TIF)

Text S12 Diagnosis of Pediatric HIV Infection Training - Diagnosis Workshop Session. A small group session to train the inpatient pediatric PITC program staff in pediatric HIV diagnosis. Includes Baylor International Pediatric AIDS Initiative facilitator and participant materials for workshop session.
(0.85 MB TIF)

Text S13 Inpatient Pediatric PITC Program - Patient Escort Training. Presentation to train patient escorts in their roles and responsibilities within the inpatient pediatric PITC program. Baylor International Pediatric AIDS Initiative PowerPoint slides 1–4.
(7.55 MB TIF)

Text S14 Inpatient Pediatric PITC Program - Patient Escort Training. Presentation to train patient escorts in their roles and responsibilities within the inpatient pediatric PITC program. Baylor International Pediatric AIDS Initiative PowerPoint slides 5-8.
(4.96 MB TIF)
