# Supplementary figures and images for: Task Shifting Routine Inpatient Pediatric HIV Testing Improves Program Outcomes in Urban Malawi: A Retrospective Observational Study
Source: PLoS One. 2010 Mar 10;5(3):e9626. doi: 10.1371/journal.pone.0009626 (PMC2835755; doi:10.1371/journal.pone.0009626)

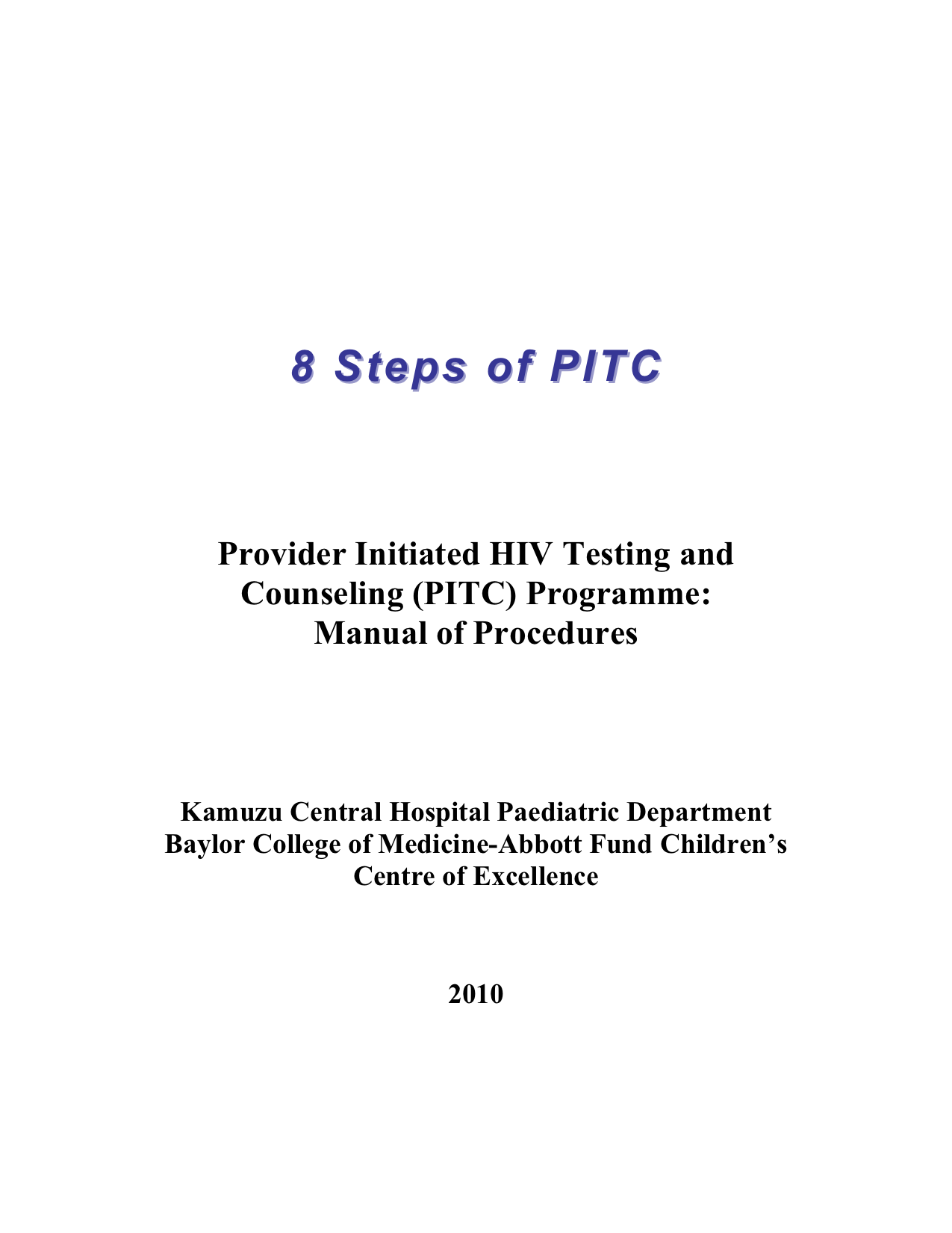

Supplement: Text S1 — Inpatient Pediatric PITC Program - Manual of Procedures. A detailed description of inpatient pediatric PITC program procedures, recommended staffing levels, suggested budget, and program materials. (8.80 MB TIF) [file pone.0009626.s001.tif]

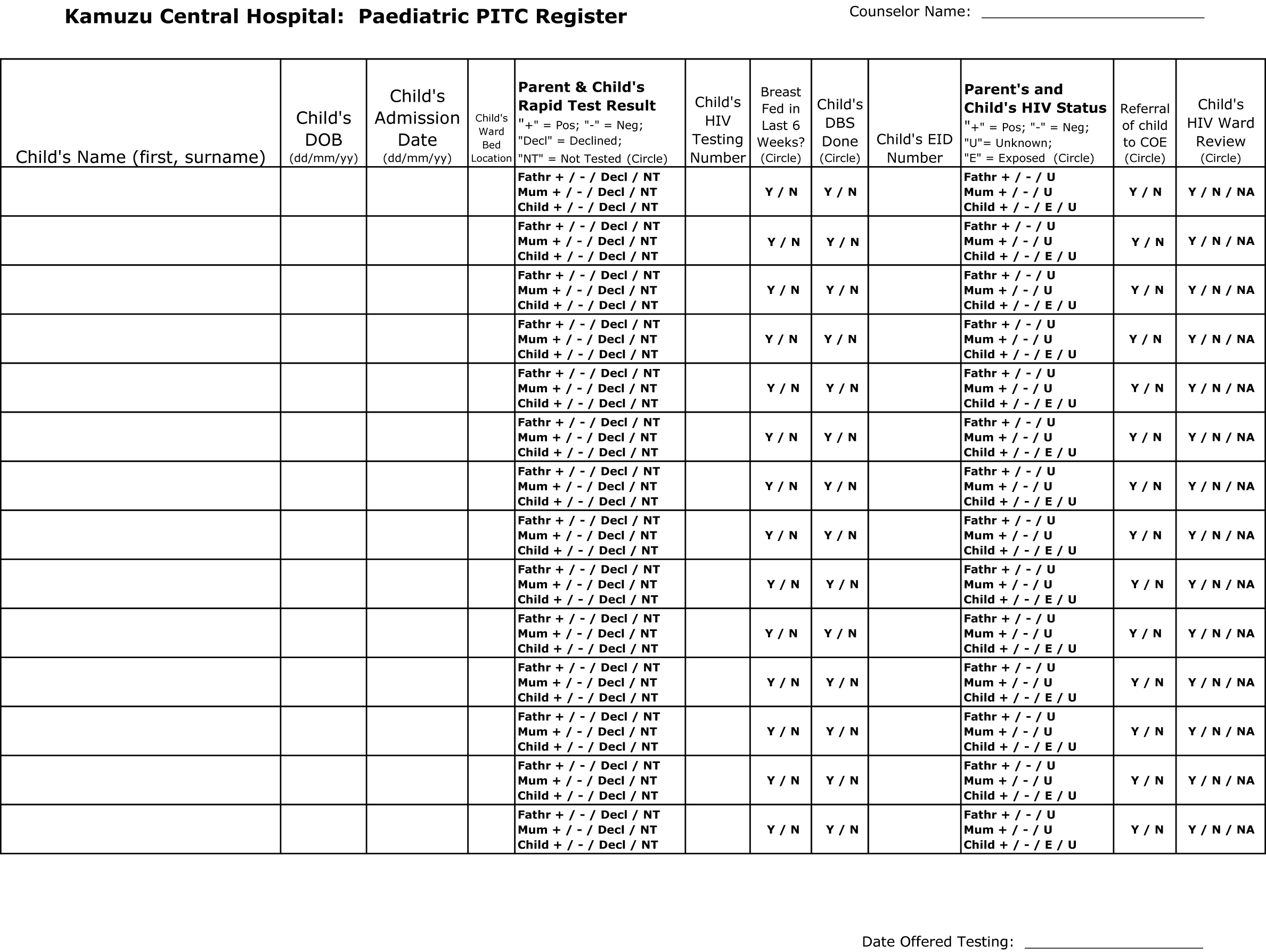

Supplement: Text S2 — Inpatient Pediatric PITC Program - Register. A confidential inpatient pediatric PITC program register for routine program monitoring and linkage of HIV-infected and HIV-exposed patients to inpatient clinical care. (1.66 MB TIF) [file pone.0009626.s002.tif]

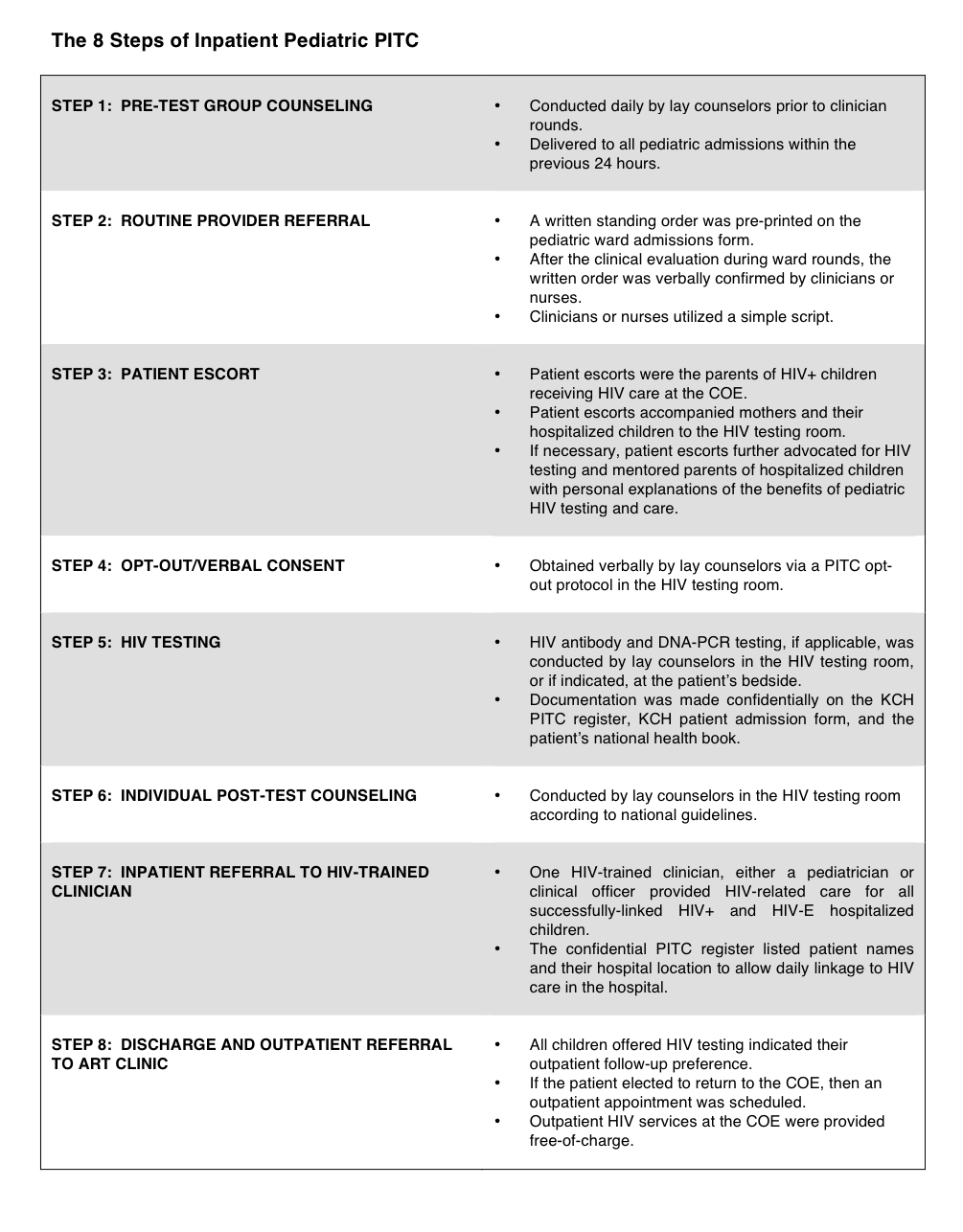

Supplement: Text S3 — Inpatient Pediatric PITC Program - Overview. A one page overview outlining the eight steps that comprise the Baylor International Pediatric AIDS Initiative inpatient pediatric PITC system. (0.26 MB TIF) [file pone.0009626.s003.tif]

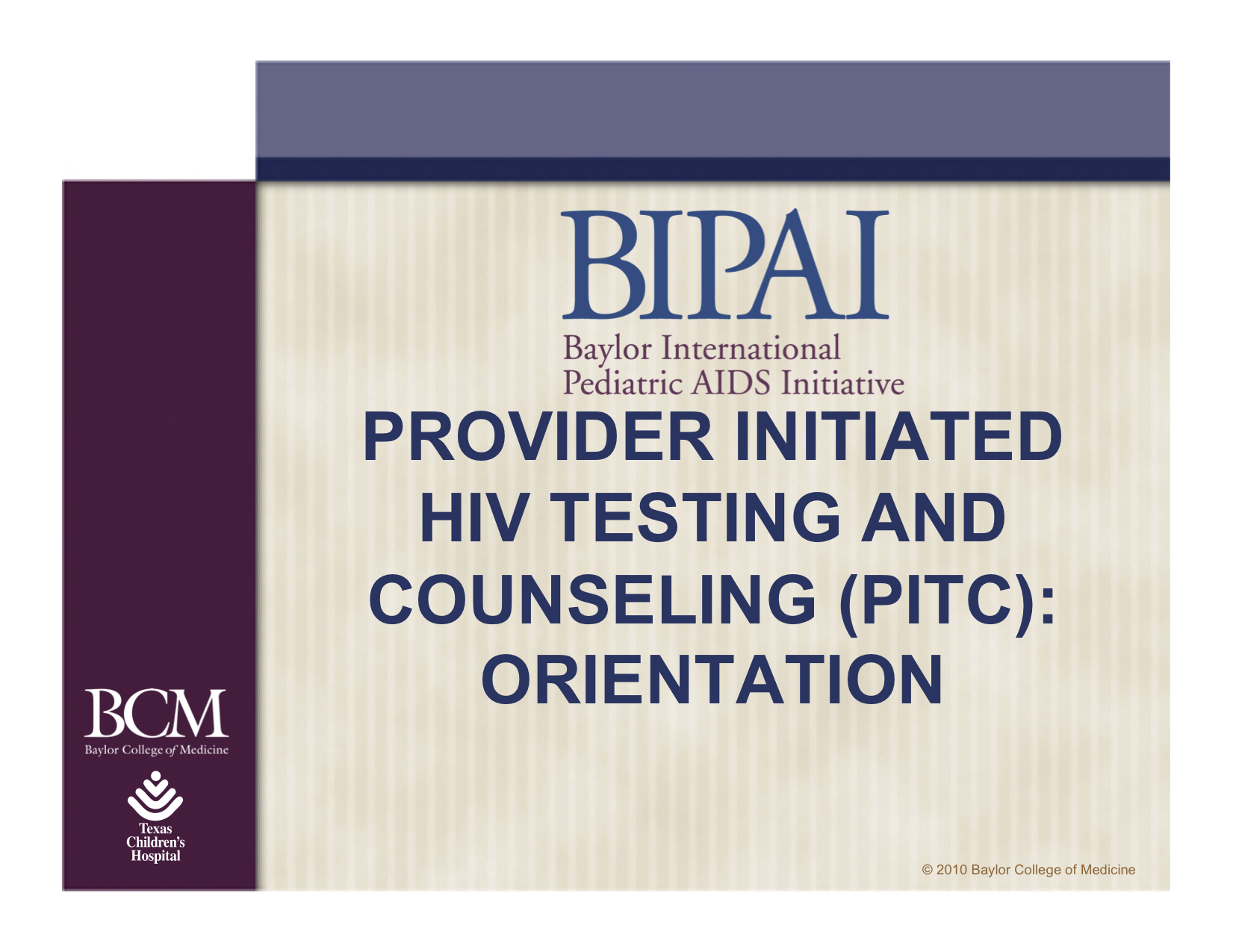

Supplement: Text S4 — Inpatient Pediatric PITC Program - Orientation. Pediatric department and inpatient pediatric PITC staff orientation presentation. Baylor International Pediatric AIDS Initiative PowerPoint slides 1–7. (9.77 MB TIF) [file pone.0009626.s004.tif]

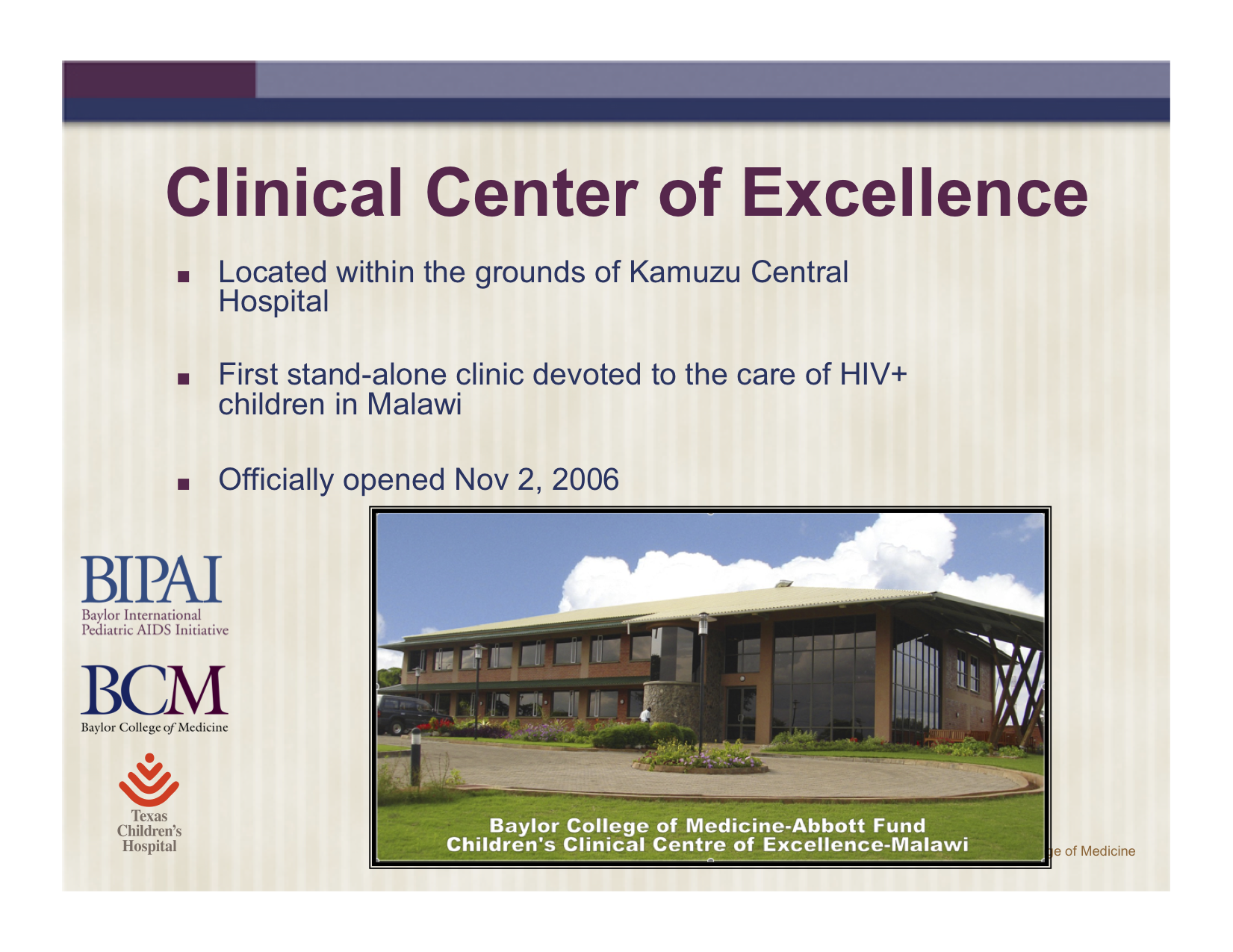

Supplement: Text S5 — Inpatient Pediatric PITC Program - Orientation. Pediatric department and inpatient pediatric PITC staff orientation presentation. Baylor International Pediatric AIDS Initiative PowerPoint slides 8–13. (9.57 MB TIF) [file pone.0009626.s005.tif]

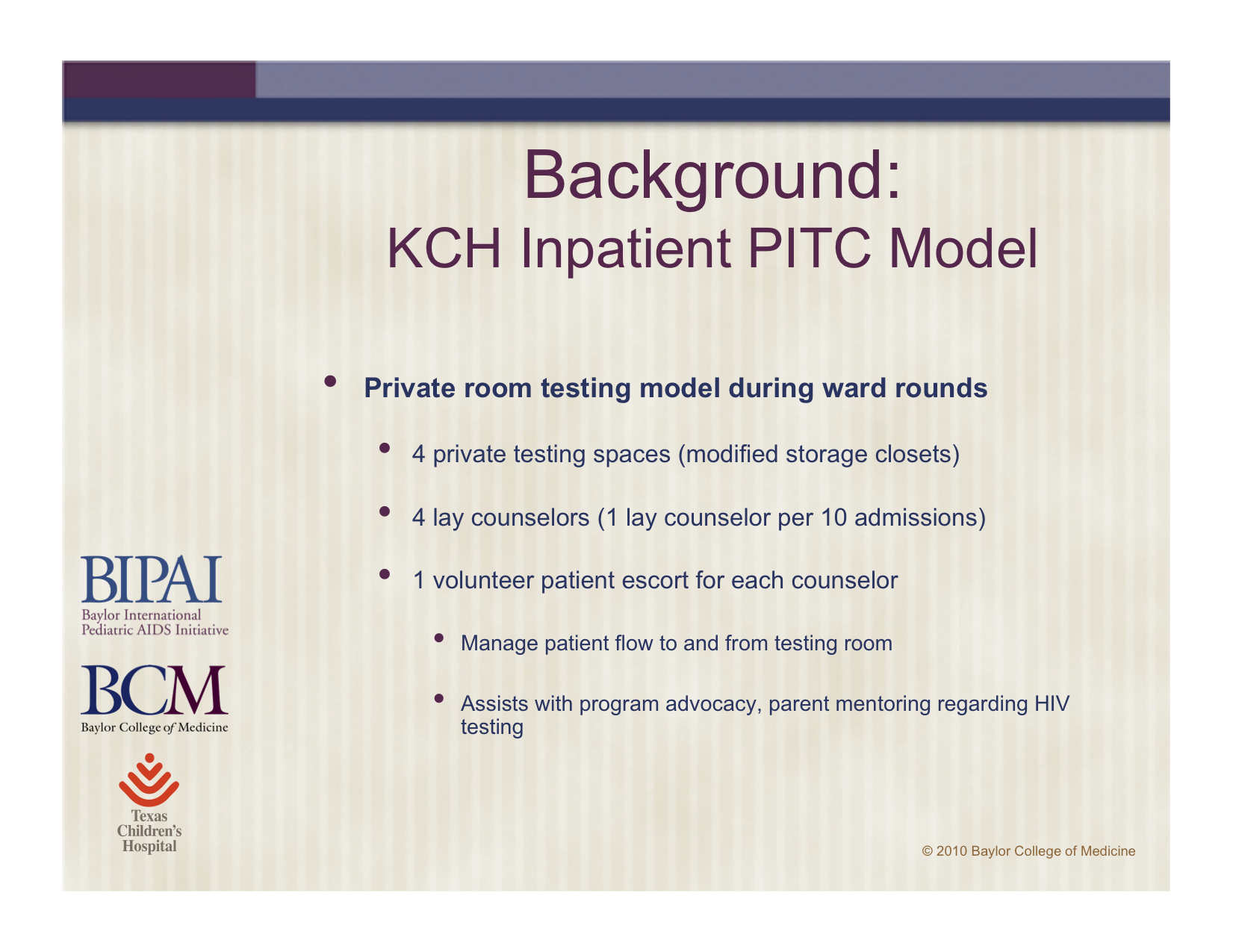

Supplement: Text S6 — Inpatient Pediatric PITC Program - Orientation. Pediatric department and inpatient pediatric PITC staff orientation presentation. Baylor International Pediatric AIDS Initiative PowerPoint slides 14–20. (8.73 MB TIF) [file pone.0009626.s006.tif]

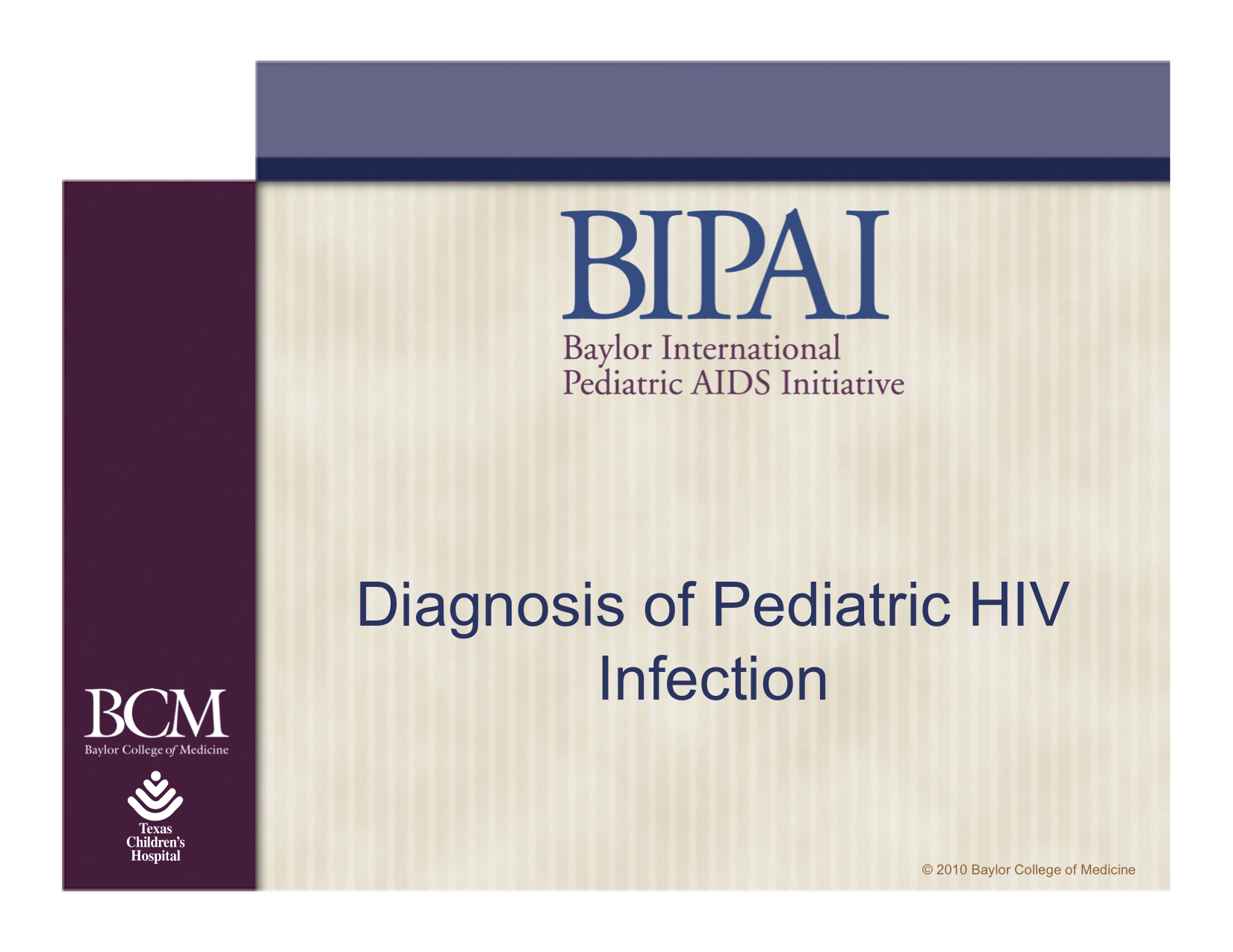

Supplement: Text S7 — Diagnosis of Pediatric HIV Infection Training. Presentation to train the inpatient pediatric PITC program staff in pediatric HIV diagnosis. Baylor International Pediatric AIDS Initiative PowerPoint slides 1–6. (7.67 MB TIF) [file pone.0009626.s007.tif]

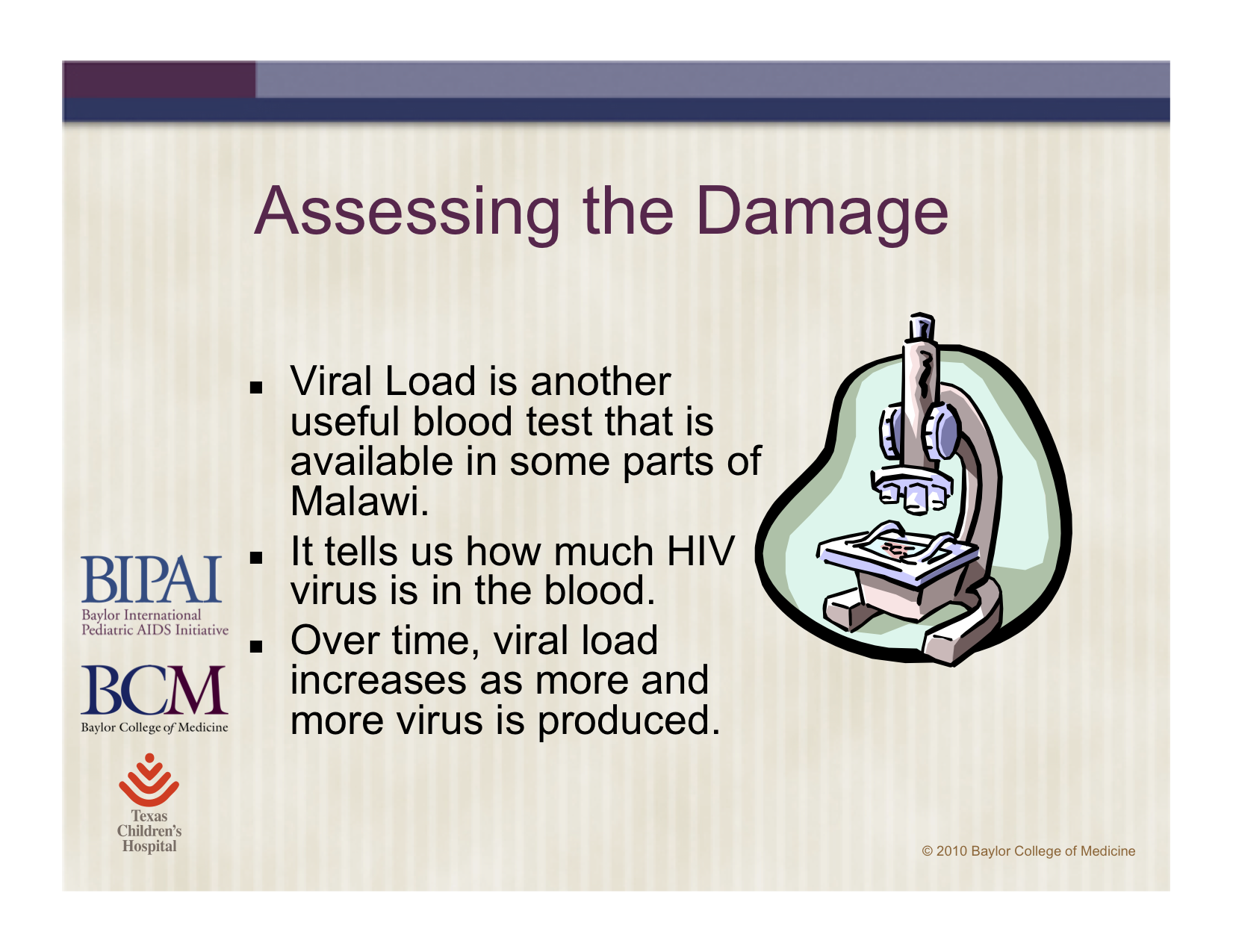

Supplement: Text S8 — Diagnosis of Pediatric HIV Infection Training. Presentation to train the inpatient pediatric PITC program staff in pediatric HIV diagnosis. Baylor International Pediatric AIDS Initiative PowerPoint slides 7–12. (6.95 MB TIF) [file pone.0009626.s008.tif]

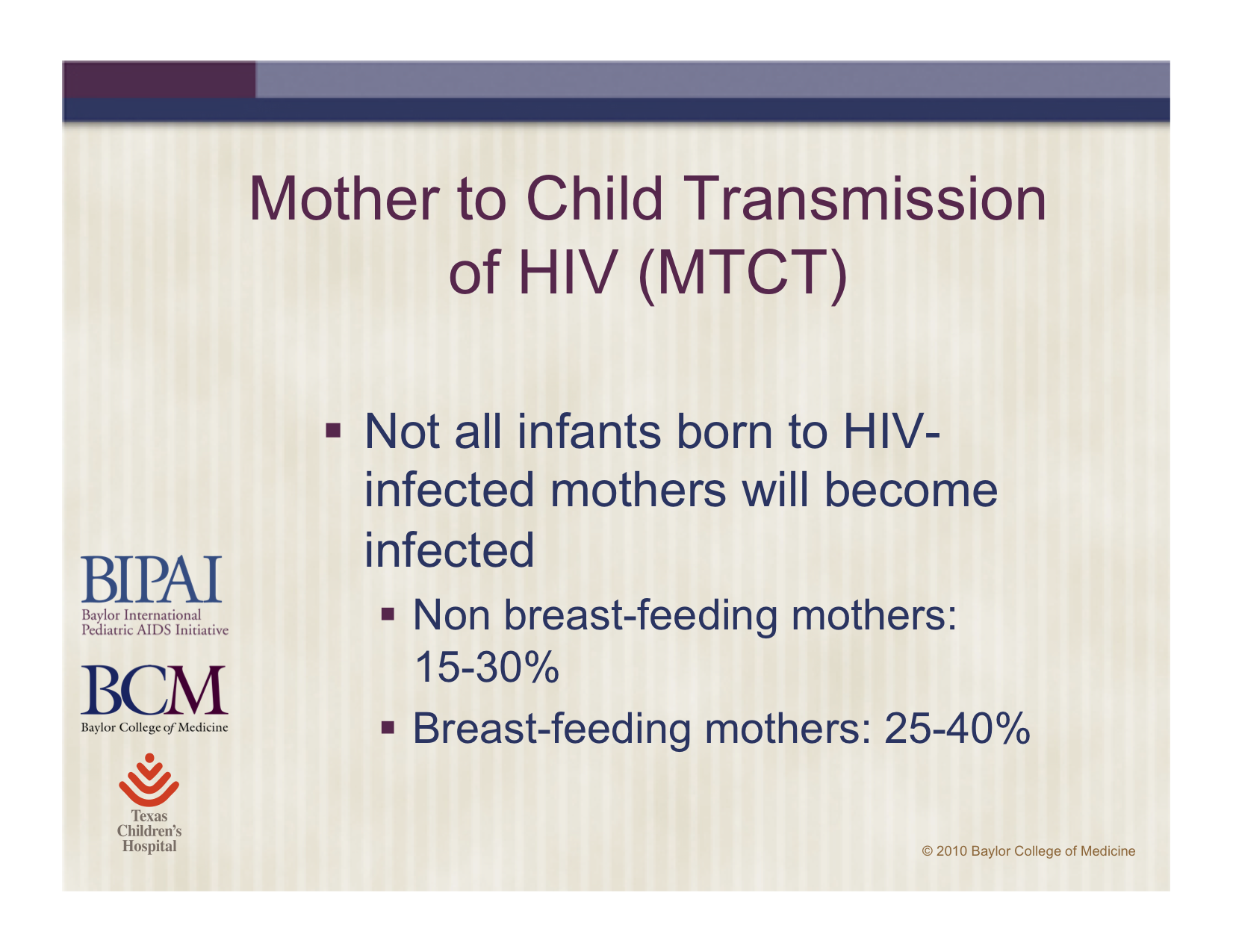

Supplement: Text S9 — Diagnosis of Pediatric HIV Infection Training. Presentation to train the inpatient pediatric PITC program staff in pediatric HIV diagnosis. Baylor International Pediatric AIDS Initiative PowerPoint slides 13–18. (7.75 MB TIF) [file pone.0009626.s009.tif]

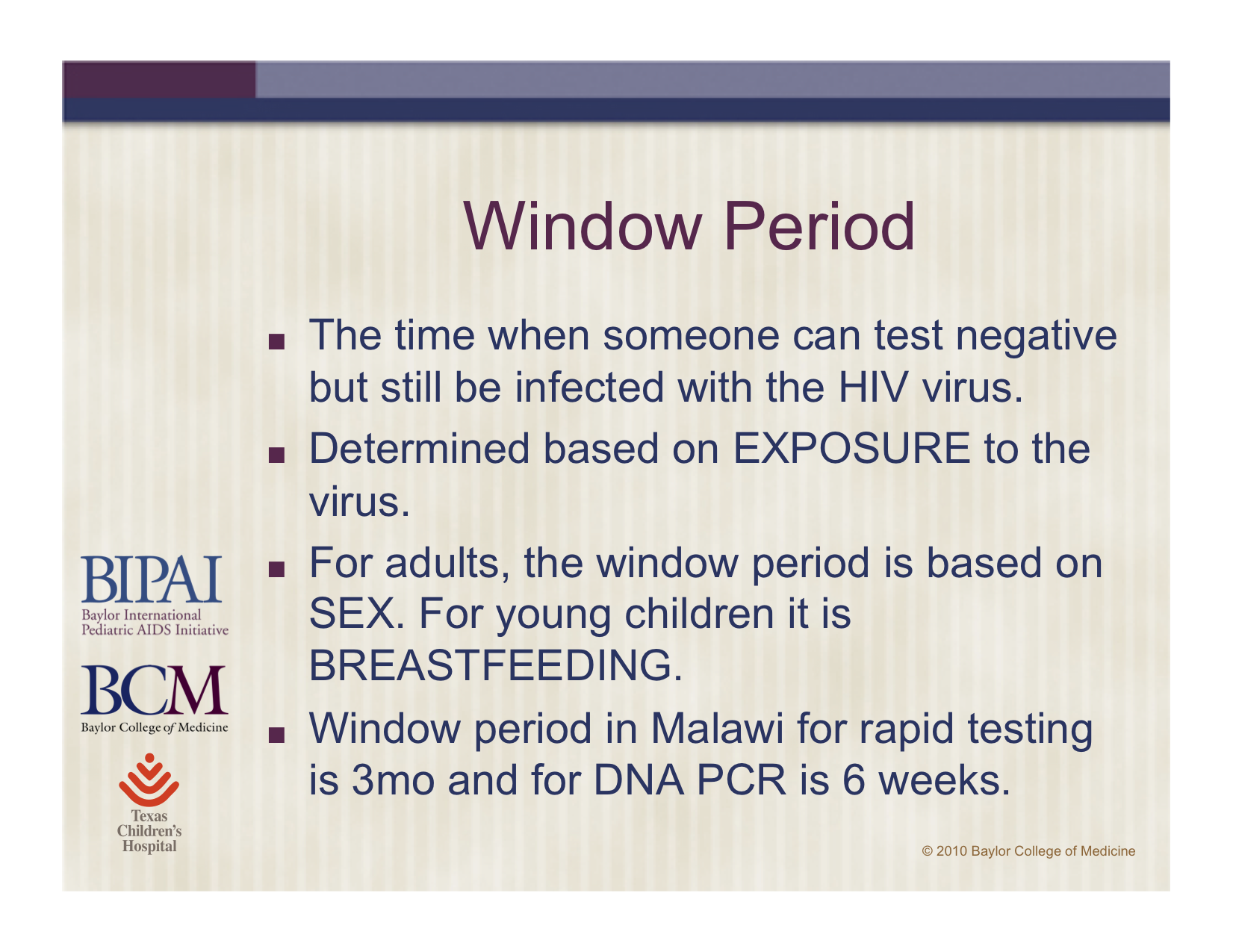

Supplement: Text S10 — Diagnosis of Pediatric HIV Infection Training. Presentation to train the inpatient pediatric PITC program staff in pediatric HIV diagnosis. Baylor International Pediatric AIDS Initiative PowerPoint slides 19–24. (7.81 MB TIF) [file pone.0009626.s010.tif]

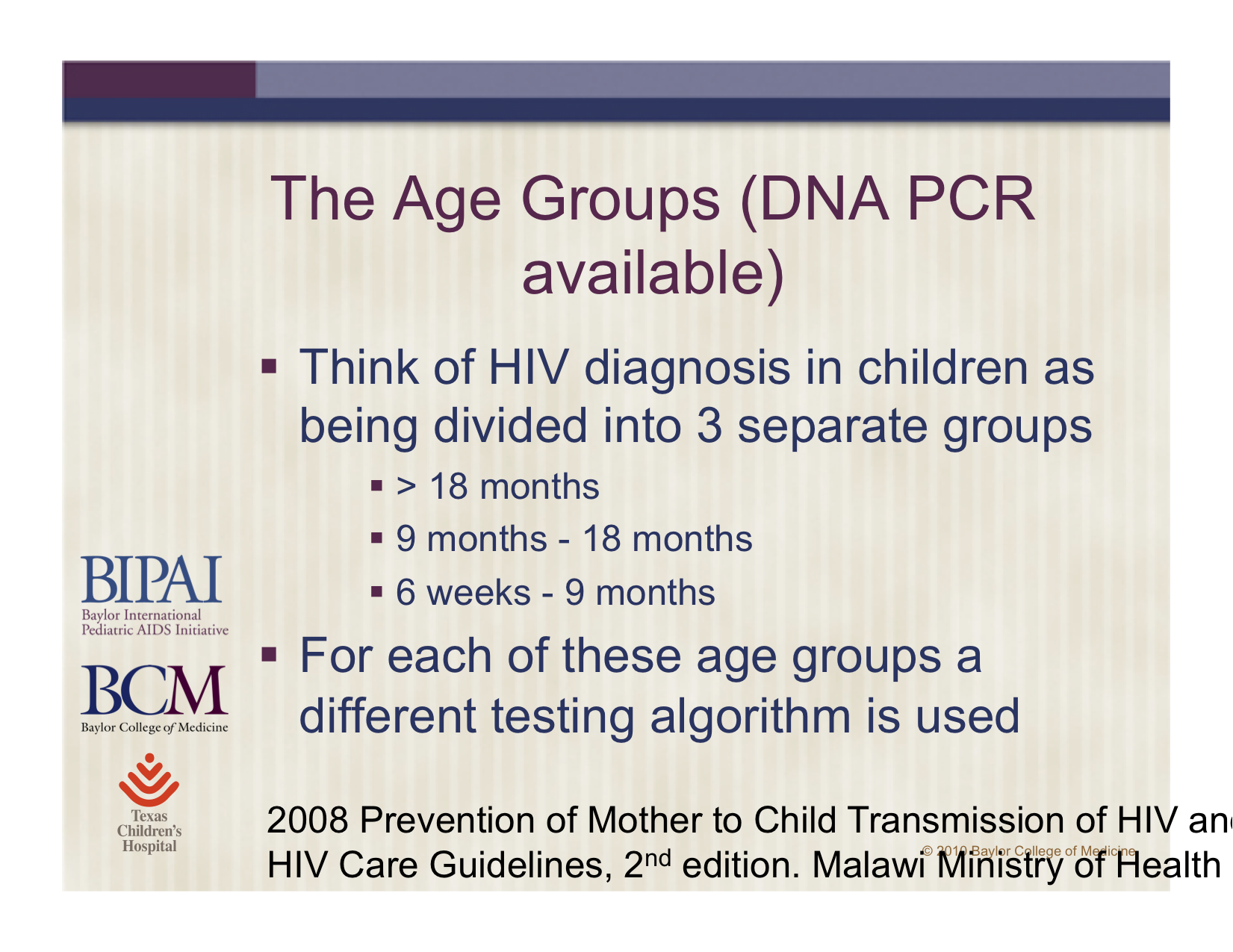

Supplement: Text S11 — Diagnosis of Pediatric HIV Infection Training. Presentation to train the inpatient pediatric PITC program staff in pediatric HIV diagnosis. Baylor International Pediatric AIDS Initiative PowerPoint slides 25–29. (6.51 MB TIF) [file pone.0009626.s011.tif]

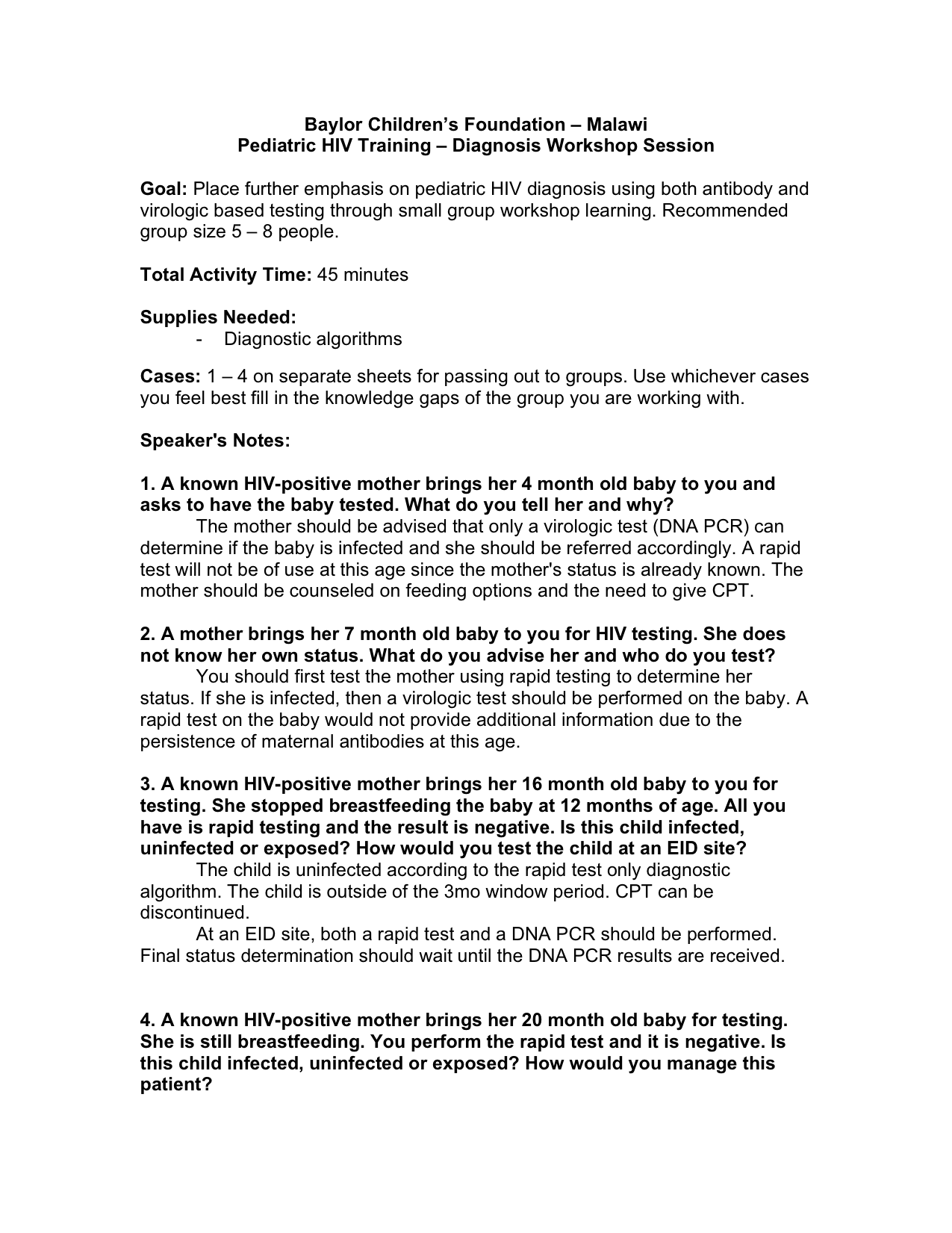

Supplement: Text S12 — Diagnosis of Pediatric HIV Infection Training - Diagnosis Workshop Session. A small group session to train the inpatient pediatric PITC program staff in pediatric HIV diagnosis. Includes Baylor International Pediatric AIDS Initiative facilitator and participant materials for workshop session. (0.85 MB TIF) [file pone.0009626.s012.tif]

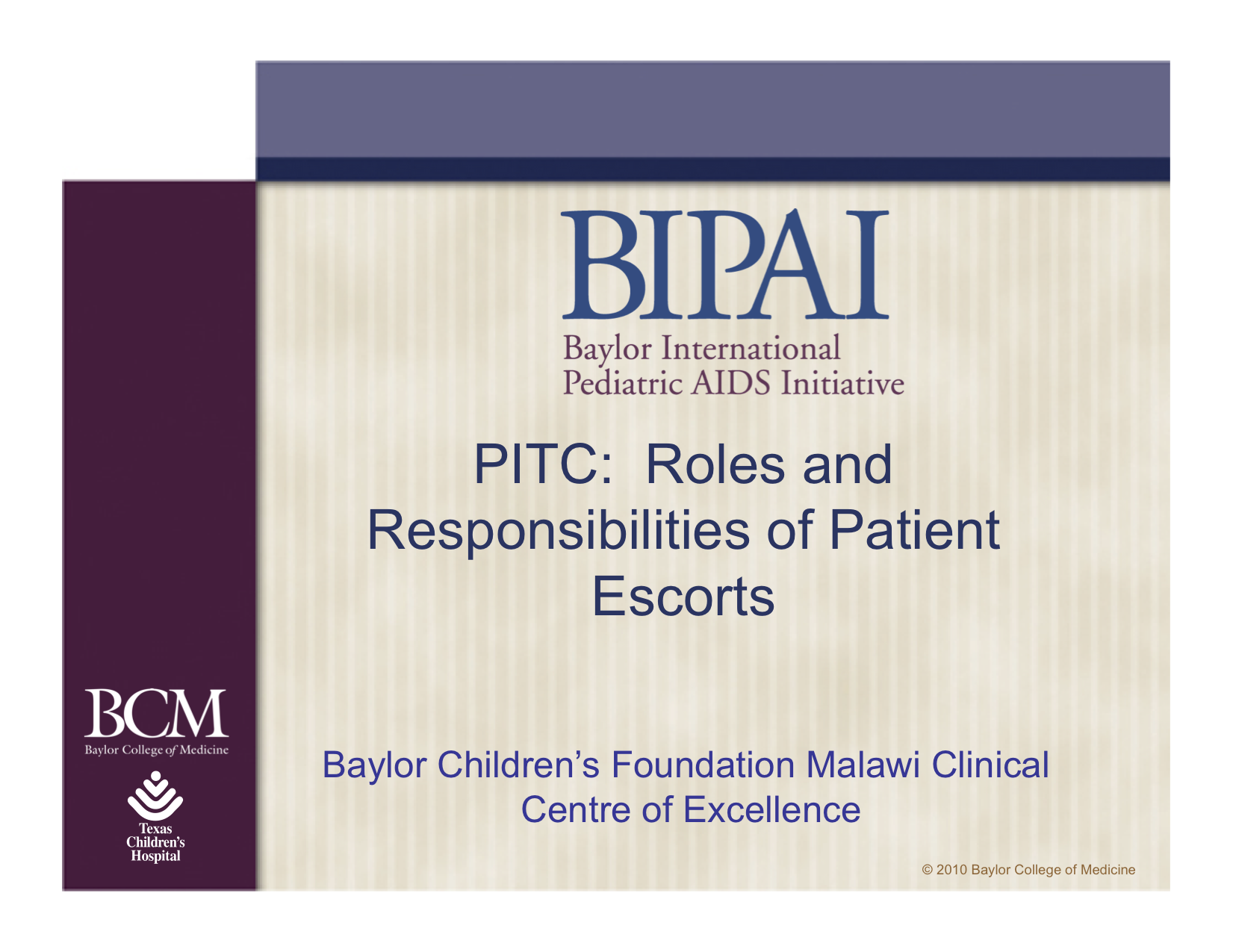

Supplement: Text S13 — Inpatient Pediatric PITC Program - Patient Escort Training. Presentation to train patient escorts in their roles and responsibilities within the inpatient pediatric PITC program. Baylor International Pediatric AIDS Initiative PowerPoint slides 1–4. (7.55 MB TIF) [file pone.0009626.s013.tif]

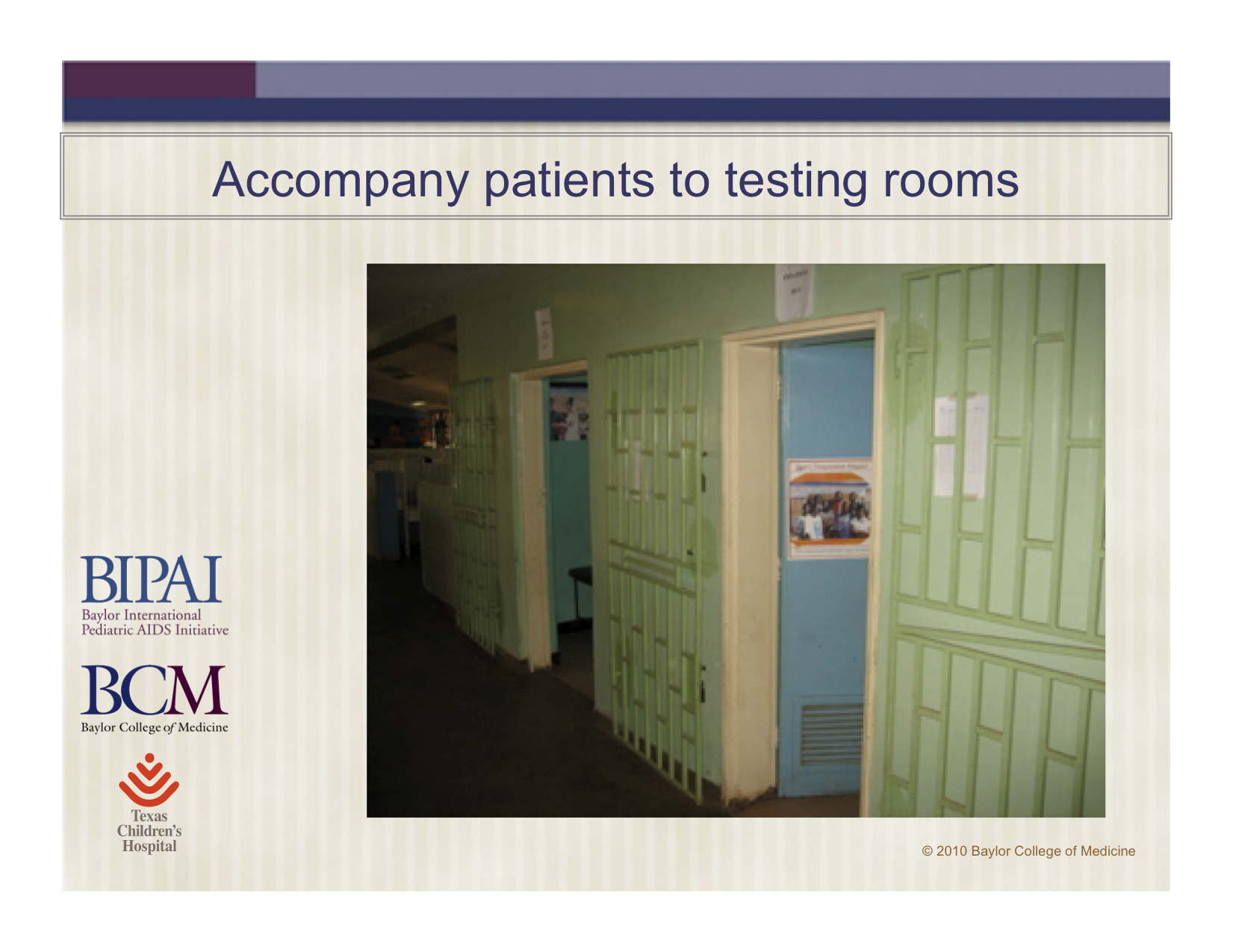

Supplement: Text S14 — Inpatient Pediatric PITC Program - Patient Escort Training. Presentation to train patient escorts in their roles and responsibilities within the inpatient pediatric PITC program. Baylor International Pediatric AIDS Initiative PowerPoint slides 5-8. (4.96 MB TIF) [file pone.0009626.s014.tif]
